# Attitudes towards Trusting Artificial Intelligence Insights and Factors to Prevent the Passive Adherence of GPs: A Pilot Study

**DOI:** 10.3390/jcm10143101

**Published:** 2021-07-14

**Authors:** Massimo Micocci, Simone Borsci, Viral Thakerar, Simon Walne, Yasmine Manshadi, Finlay Edridge, Daniel Mullarkey, Peter Buckle, George B. Hanna

**Affiliations:** 1 NIHR London In-Vitro Diagnostics Cooperative, London W2 1PE, UK; s.borsci@imperial.ac.uk (S.B.); s.walne@imperial.ac.uk (S.W.); p.buckle@imperial.ac.uk (P.B.); g.hanna@imperial.ac.uk (G.B.H.); 2 Department of Surgery and Cancer, Imperial College London, London W2 1PE, UK; 3 Faculty of Behavioural, Management and Social Sciences (BMS), University of Twente, 7522 NB Enschede, The Netherlands; 4 Department of Primary Care and Public Health, Imperial College London, London W6 8RP, UK; v.thakerar@imperial.ac.uk; 5 Skin Analytics Limited, London EC2A 4PS, UK; yasmine@skinanalytics.co.uk (Y.M.); finlay@skinanalytics.co.uk (F.E.); dan@skinanalytics.co.uk (D.M.)

**Keywords:** artificial intelligence, trust, passive adherence, human factors

## Abstract

Artificial Intelligence (AI) systems could improve system efficiency by supporting clinicians in making appropriate referrals. However, they are imperfect by nature and misdiagnoses, if not correctly identified, can have consequences for patient care. In this paper, findings from an online survey are presented to understand the aptitude of GPs (*n* = 50) in appropriately trusting or not trusting the output of a fictitious AI-based decision support tool when assessing skin lesions, and to identify which individual characteristics could make GPs less prone to adhere to erroneous diagnostics results. The findings suggest that, when the AI was correct, the GPs’ ability to correctly diagnose a skin lesion significantly improved after receiving correct AI information, from 73.6% to 86.8% (X^2^ (1, *N* = 50) = 21.787, *p* < 0.001), with significant effects for both the benign (X^2^ (1, *N* = 50) = 21, *p* < 0.001) and malignant cases (X^2^ (1, *N* = 50) = 4.654, *p* = 0.031). However, when the AI provided erroneous information, only 10% of the GPs were able to correctly disagree with the indication of the AI in terms of diagnosis (d-AIW M: 0.12, SD: 0.37), and only 14% of participants were able to correctly decide the management plan despite the AI insights (d-AIW M:0.12, SD: 0.32). The analysis of the difference between groups in terms of individual characteristics suggested that GPs with domain knowledge in dermatology were better at rejecting the wrong insights from AI.

## 1. Introduction

Artificial Intelligence (AI)-based technologies used for medical purposes may have the ability to change the healthcare landscape, providing opportunities for the prioritization of patients who are most at risk [1] and for the support of clinicians making diagnostic conclusions [2]. 

A growing field of development of AI systems is dermatology, in which early detection of melanoma may benefit patients [3,4,5]. Every year in the UK, General Practitioners (GPs) see over 13 million patients for dermatological concerns [6]; melanoma is one of the most dangerous forms of skin cancer, with the potential to metastasise to other parts of the body via the lymphatic system and bloodstream. The current standard of care for skin cancer is set by the National Institute for Health and Care Excellence (NICE) [7], which adopt a ‘risk threshold’ value of 3% positive predictive value (PPV) in primary care to underpin recommendations for suspected skin cancer pathway referrals and urgent direct access investigations in cancer. GPs are expected to refer under the 2WW if the probability of cancer is 3% or higher. Referral rates are also influenced by factors beyond clinical suspicion of the lesion, such as a clinician’s individual risk tolerance and perceived patient expectations or concerns [8]. Dermatology is the speciality with the highest referral rate in the NHS [9]; however, of the half a million cases referred on this pathway, melanoma and squamous cell carcinoma (SCC) only made up 6.5% of referrals in 2019/20 [10]. This reflects the accepted behaviour amongst clinicians of referring with a very low threshold to facilitate detection in the early stages of the disease. The same data from the National Cancer Registration and Analysis Service (NCRAS) also indicate that only 64% of cancers are detected through 2WW referrals, suggesting that considerable numbers of skin cancer cases are detected through alternative pathways, potentially representing missed diagnoses by GPs and risking delays in diagnosis. These professionals, given their role as generalists rather than specialist dermatologists [11], represent the first line of defence against skin cancer, and they might benefit from the support of an accurate AI solution for the early detection of skin cancer and the identification of atypical presentations, with an overall beneficial impact for patients and the NHS [12].

The number of studies assessing the efficacy of intelligent systems for dermatology applications [13,14,15,16,17,18] is significant. However, to date, only a few of these AI-enabled medical devices have made it through to real-world deployment. This is also a result of a lack of randomized trials [18] and the absence of AI assessments for lesions with abnormal presentation and clinical features similar to melanoma that may produce erroneous diagnoses [19]. These tools are dependent on the quantity and quality of training data [12,20]. The introduction of algorithm-based tools into a complex socio-technical system may create friction and conflict in decision making; this is due to the intrinsic tendency of artificial intelligence to reach a certain ‘conclusion’ that may not be transparent to human decision-makers and the consequent alterations in practices. 

Ultimately, the key issue with AI is how much decision makers will trust these medical devices once deployed in the market. The inclusion of AI systems in the healthcare field should be supported by the awareness that these systems, like the existing workforce, are imperfect. For decision support tools, the resilience of the diagnostic process is in the hands of the clinicians, even when an AI is involved, as they are the only ones who have a holistic view of each clinical scenario, and they can decide to agree or disagree with an AI [21]. Beyond the issue associated with having a ‘black box’ AI or a fully transparent tool to support decisions [22], the main risk could also be that professionals might over-trust the insights provided by these tools due to a lack of expertise in the use of the technology or the complexity around the cases [4,23,24]. 

In this paper, we present results from an online survey conducted on a pool of GPs who were presented with a combination of accurate and inaccurate results from a hypothetical AI-enabled diagnostic tool for the early detection of skin cancer. This study aimed to explore the attitudes of GPs when asked to trust (or not to trust) the AI diagnosis as appropriate. We also explore ‘predicting factors to trust’ that would make GPs resilient enough to prioritise their clinical opinion when an AI produces erroneous diagnoses. 

## 2. Materials and Methods

A total of 73 GPs participated in the study. Among them, 23 were excluded because they were not able to finalise or correctly complete the test. The final sample of 50 GPs (mean age: 34.4, min = 26, max = 53; 76% female) completed the test online via Qualtrics^XM^ between the 10 April 2020 and the 10 May 2020. Participants were directly informed of this study and recruited by email through a clinical lead in primary care research at the NIHR LIVD; also, the link to the survey was posted on social media (Twitter and LinkedIn) and in a private WhatsApp group used by GPs and GPs with special interests working in the Greater London area. 

The online test was composed of the following sections:

- Demographics. This section was composed of 15 items. It included qualitative questions regarding individual characteristics (age, gender, years of practice etc.) and questions regarding the respondent’s interest in dermatology and attendance at dermatology courses in the past three years, as well as their perceived confidence in dermatology and familiarity with tools for early skin cancer diagnosis. Three questions considered the GPs overall trust attitude toward innovations in medical devices [25].
- Main test. This was composed of questions on 10 lesions (See Appendix A) purposively selected to be representative of commonly encountered lesions. The cases presented are realistic. Cases of misclassification were modified to explore GPs’ attitudes when their diagnosis conflicted with those from the AI.

Each lesion was accompanied by vignettes of hypothetical patient details likely to be asked after in a routine GP consultation (age, gender, duration of the skin lesion, evolution/changes of the lesion, sensory changes, bleeding, risk factors, body location). Each lesion was associated with three questions pertaining to:

- The diagnosis, with a range of seven options (melanoma; squamous cell carcinoma; basal cell carcinoma; intra-epidermal carcinoma; actinic keratosis; benign, other);
- The management plan, with a range of four options (two-week 2WW referral; routine, but not 2WW; discharge with safety net advice; other);
- The confidence in their decision making, on a five-point Likert scale.

The 10 skin lesions were divided in terms of the type of decision making and type of case (benign and malignant) as follows:

- Everyday cases (EC-5 lesions), including lesions whose features are commonly observed in routine consultations and considered easy to interpret [26]; two of these were benign and three were malignant skin lesions (cases 2–6);
- Cases with uncertainties (CU-3 lesions); i.e., cases in which the picture of the skin lesion is hard to interpret or it contains a bias (marked for biopsy) and for which GPs might be expected to ask for a second opinion. One of these CU cases was malignant and two were benign (cases 1, 7 and 8). For all the cases from 1 to 8 (EC and CU), the scenario was set up with the AI system presenting the correct diagnosis to the GPs;
- Dangerous scenarios (DS-2 lesions), including one benign case misclassified as malignant and one malignant case misclassified as benign.

### 2.1. Procedure

**Figure 1:**
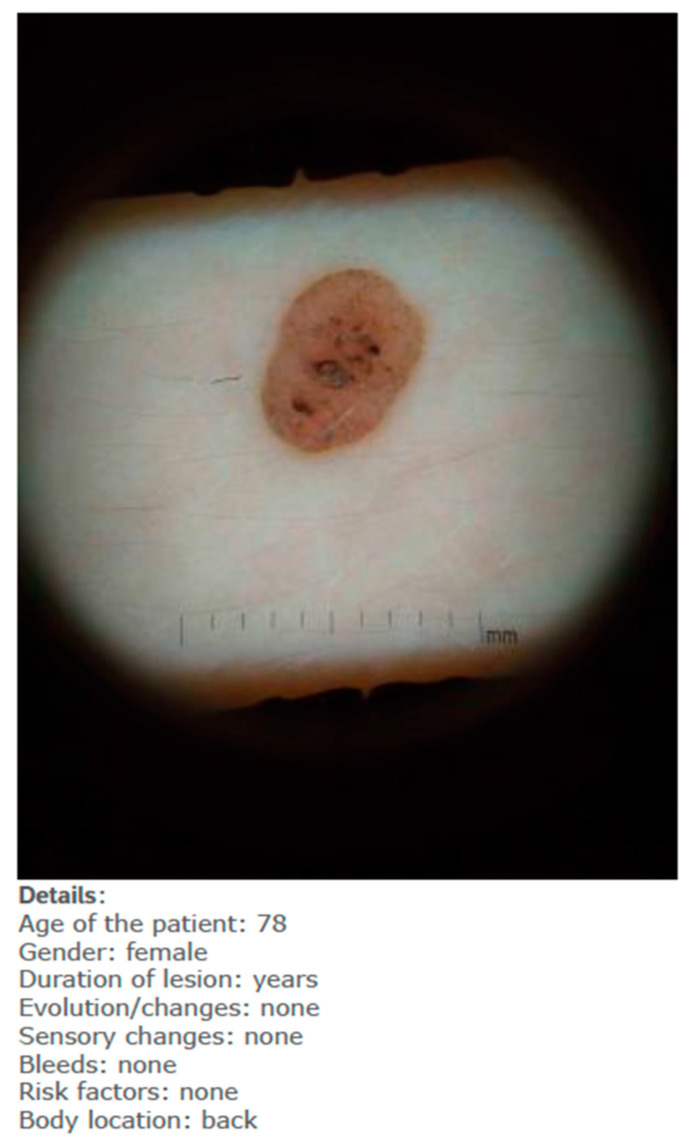
Example of one lesion with only patient information (fictitious).

**Figure 2:**
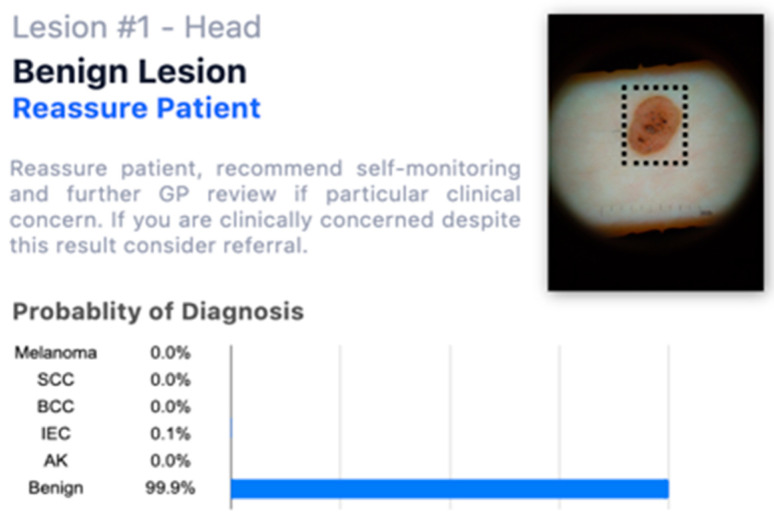
Example of one lesion with a fictitious AI assessment.

The study was presented to participants as a simulation—with fictitious patients’ details—to assess their agreement with an AI system to better report diagnostic test results. Once the study was completed, a disclaimer email was sent to each participant clarifying that the provided combinations of lesions/diagnoses in the study were not always accurate; the study aim of assessing GPs’ performance and attitudes with both accurate and inaccurate AI diagnoses was fully explained. After the demographic survey, each participant received ten blocks of questions (each related to one lesion) in a fully randomised order. Participants completed these questions regarding the diagnosis, the management plan and their confidence twice:

1. When they had access only to patient information and images of the skin lesions (Figure 1);
2. When they had access to the AI insights, as shown in Figure 2, in addition to this information;

GPs were then asked to decide whether to change or to maintain their answers regarding the diagnosis, management plan and their confidence in their decision.

### 2.2. Data Analysis 

Descriptive statistics were used to observe participants’ characteristics, the frequency of correct diagnoses and management plans, and the GPs’ confidence in their decision making before and after receiving the AI-enabled information. The pre-and post-AI performance levels of the GPs, in terms of their diagnoses and management plans, were dichotomised (correct/incorrect) and McNemar’s Chi-square test was used to analyse the effect of AI information in each decision-making group (EC, UC, DS) by also accounting for the type of case (benign and malignant). The percentage of confidence was tested using a generalized linear mixed model. 

The hit and false rates of the GPs for the diagnostic and management decision making before and after the wrong AI insights were used to model GPs’ resilience when dealing with erroneous AI information (i.e., DS cases). In line with signal detection theory [27], a computation was used to compose a sensitivity index for when AI was wrong (d-AIW, see Appendix B); the higher the index compared to zero, the better the GP’s ability to ignore the wrong indication of the AI. The index was used to distinguish two groups: one included GPs who had a d-AIW over zero (hereafter called the ‘resilient group’) and the other included GPs with an index below or equal to zero (hereafter called the ‘non-resilient group’) for the management and diagnostics of patients with skin lesions. A Kruskal–Wallis test was performed to check if resilient and non-resilient GPs performed significantly differently when AI provided them with correct and incorrect answers and to observe the differences between the two groups in terms of individual characteristics.

## 3. Results

### 3.1. Individual Characteristics 

In total, 76% of the participants had less than 5 years of experience, 16% from 5 to 10 years and 8% had more than 10 years of experience. Overall, the GPs in our cohort declared an average level of confidence in dermatology of 51.5 out of 100 (SD: 16.2), although 34% of them had attended specialisation courses on the topic in the previous three years. Seventy per cent of the participants stated that they had not used a dermatoscope in the previous 12 months, with only 4% of the GPs declaring weekly use of such an instrument. Thirty-eight per cent never used digital systems for skin lesions (e.g., taking pictures of patients’ skin lesions to be uploaded into the system), while among those who used such digital systems for diagnostic purposes, 2% declared daily usage, 10% weekly and 50% stated that they used them at least once per month. The level of trust toward AI support systems declared by GPs for this application domain was sufficient (M: 61.2%; SD: 14.5%).

### 3.2. General Practitioners’ Correct Decision Making before and after AI Insights

Table 1 shows the statistics of GPs’ performances before and after receiving the fictitious AI-enabled information, which suggests that GPs tended to adhere to the indications of the AI. Specifically, when the AI was correct (EC and CU cases), there was a positive effect on GPs’ performance and confidence. Correct diagnosis, supported by a trustworthy AI, went up by 13.2 points for EC cases and 16.5 points for CU cases. Similarly, the selection of the correct management plan went up by 7.6 points (EC) and 18.5 points (CU). GPs’ confidence in their decision making went up of 12.7 for EC cases after the insights of the AI, while this aspect only increased by 1.5 points when dealing with CU cases. Conversely, when the AI provided incorrect insights (DS cases), the correctness of diagnoses and management went down by 24 and 29 points respectively, with a positive boost of 5.7 points in the GPs’ confidence in their decision making after receiving AI insights. 

McNemar’s Chi-square test clarified how the AI insights affected the GPs’ decision making for each group.

Everyday cases: GPs’ ability to correctly diagnose a skin lesion significantly improved after receiving the AI information from 73.6% to 86.8% (X^2^ (1, *N* = 50) = 21.787, *p* < 0.001), with significant effects for both the benign (X^2^ (1, *N* = 50) = 21, *p* < 0.001) and malignant (X^2^ (1, *N* = 50) = 4.654, *p* = 0.031) cases. The selection of the correct management plan was also positively affected by the AI information, going from 82.4% to 90% (X^2^ (1, *N* = 50) = 3.78, *p* < 0.001), and it was particularly relevant for the plans regarding benign cases (X^2^ (1, *N* = 50) = 22, *p* < 0.001), while no major improvement was observed for malignant cases. Confidence about decision making, independent of the type of skin lesion, significantly improved from 66.8% to 79.5% after receiving the AI information (X^2^ (1, *N* = 48) = 107.2, *p* < 0.001).

Cases with uncertainties (CU): GPs’ correct diagnosis improved significantly from 37.5% to 54% correct decision making when supported by the AI (X^2^ (1, *N* = 50) = 24.9, *p* < 0.001). This difference was significant for benign cases (X^2^ (1, *N* = 50) = 31.03, *p* < 0.001), while no significant differences emerged in malignant cases before and after receiving AI information. Concurrently, the ability to correctly define a management plan significantly increased from 44% to 62.5% thanks to the AI (X^2^ (1, *N* = 50) = 28.195, *p* < 0.001), and this effect was significant for begin cases (X^2^ (1, *N* = 50) = 31, *p* < 0.001). GPs’ confidence was not significantly affected by the AI information.

Dangerous situations (DS): When erroneous information was provided by the AI, it seems that GPs were significantly pushed to adhere to the erroneous suggestions of the AI. Correct diagnosis of the skin lesions significantly decreased from 46% to 22% (X^2^ (1, *N* = 50) = 22.04, *p* < 0.001). Adherence to the wrong AI insights was significant for both benign (X^2^ (1, *N* = 50) = 9.08, *p* = 0.026) and malignant (X^2^ (1, *N* = 50) = 11.7, *p* = 0.009) cases. Similarly, decision making about management was significantly affected by wrong AI insights, decreasing the ability of GPs to correctly decide the plan for the patient from 54% to 25% (X^2^ (1, *N* = 50) = 25.290, *p* < 0.001). This significantly affected GPs’ decision making regarding both benign (X^2^ (1, *N* = 50) = 12.07, *p* = 0.005) and malignant (X^2^ (1, *N* = 50) = 11.52, *p* = 0.007) cases. Confidence was not affected by the information provided by the AI.

### 3.3. Resilience to the Erroneous Insights of the Artificial Agent

When the AI provided erroneous information (DS cases), only 10% of the GPs were able to correctly disagree with the indication of the AI in terms of diagnosis (d-AIW M: 0.12, SD: 0.37), and only 14% of participants were able to correctly decide the management plan despite the AI insights (d-AIW M: 0.12, SD: 0.32). These GPs were categorized as the resilient ones (i.e., the ones able to correctly reject the AI insights), as opposed to all the others, who were categorized as less resilient to the wrong indications of the AI.

The Kruskal–Wallis test, when carried out on EC and CU cases (when the AI provided correct results), suggested that the performance of the GPs in the resilient group was not significantly different to the performance of the less resilient group. Conversely, when the AI provided erroneous diagnoses (DS cases), a significant difference was found between the two groups in terms of diagnostic decision making (X^2^ = 12.4, *p* < 0.001) and the correct management plan (X^2^ = 6.8, *p* = 0.009). 

The analysis of the differences between the groups in terms of individual characteristics suggested that GPs who declared regular usage of the dermatoscope were better at rejecting the wrong insights from the AI and making correct diagnoses (X^2^ = 7.8, *p* = 0.005) and at managing patients (X^2^ = 5.1, *p* = 0.023) compared to less resilient GPs. Some moderate but still significant effects also emerged concerning GPs’ overall confidence in dermatology, indicating that resilient GPs were more confident than non-resilient doctors, and this may have played a role in their ability to correctly diagnose (X^2^ = 3.8, *p* = 0.049) and define a management plan (X^2^ = 5, *p* = 0.024) even when the AI provided erroneous insights. The other individual factors (e.g., age, sex, training, predisposition to trust, etc.) only showed some moderate tendencies.

## 4. Discussion

The results demonstrate high levels of trust among GPs towards results attributed to a fictitious AI system, a finding which has both positive and negative implications for the healthcare system. Whilst an accurate clinical decision support tool may support GPs in correctly identifying benign lesions, thus reducing the number of false positives referred to 2WW clinics, there is also a possibility that an erroneous result from the AI system could lead to a patient’s case being under-triaged.

Adherence to an AI system that can provide correct insights about cases, even when there are uncertainties, can significantly improve the decision making (diagnosis and plan) of GPs. The correctness and confidence of GPs in their decision making were significantly improved by using the AI when a case presented no uncertainties. Given the pressure on the 2WW pathway, this result may be convenient for ruling out negative cases at the triage stage, with benefits on patient flow and for the individual patients who will avoid unnecessary anxiety associated with a suspected cancer referral. However, when dealing with some uncertainties (CU cases) or when the AI was wrong (DS cases), the confidence of the GPs in the final decision was not affected by the AI insights. This might suggest that when GPs had doubts on how to treat a case (CU cases) or when they were not convinced by the insights of the AI (DS cases), they were not completely reassured by the use of the AI; however, a large majority of the GPs continued to adhere to the indications of the AI. These findings are in alignment with previous studies [28] suggesting that over-reliance on automated systems may be triggered by confirmatory bias when participants direct their attention towards features consistent with the (inaccurate) advice. We also considered the variability of personal expertise and attitudes towards automated systems as having an influence by reducing passive adherence. The results suggest that the tendency to adhere, even when the AI is inaccurate, may be due to a lack of experience with the specific tasks or domain knowledge that may bring GPs to overestimate the insights of the intelligent systems. The small number of resilient GPs who were able to critically interpret the results of the AI declared significantly higher usage of essential dermatological tools (i.e., dermatoscope) and confidence in the specific domain of dermatology compared to the GPs who adhered to the suggestions of the mistaken AI. 

The present pilot study is intended as an initial step in the understanding of the future relationship between AI and clinicians in the domain of dermatology. 

### Limitations and Future Work

Three main limitations of the present work should be considered for future studies. 

First, the small sample surveyed may not be representative of the variety of expertise, exposure to dermatology cases and experience with similar technologies that GPs may have. A power analysis using SAS revealed a 95.9% power to detect the difference in correctness with and without AI support. Our sample size could have detected a minimum difference of 6.5% with 80% power. 

Secondly, the participants of the present study were aware that the test was a simulation and that no real AI technology was involved; therefore, we cannot rule out that they may have changed their behaviour because of the attention they received [29] and because of the absence of implications for patients. This effect may have implications for the generalisability of our findings. 

Finally, how information from an AI system is presented may impact the end-user. In future studies, we advocate a larger group of GPs, with different expertise, varying familiarity with AI systems, and different cultural backgrounds to expand the current results. Concurrently, a larger number of cases should be tested with equal numbers of different types of lesions in each group. This may bring further insights into the mechanism that leads to adherence to information provided by AI. Mixed-methods studies [30] could help in mitigating the effects of bias and changes in the behaviour of research participants under the influence of observation and measurement. The risk of a passive adherence to AI in the real world could also emerge due to the complexity of the healthcare system [21] and future longitudinal studies on real cases should be implemented to monitor such a possibility. As well as the user interface, the role of training and documentation, such as the ‘Instructions for Use’ (IFU), should be considered in future research, both academically and from the perspective of regulatory applications. 

## 5. Conclusions

Well-designed, accurate and intelligent systems may be able to support GPs in managing patients in primary care with suspicious skin lesions confidently and appropriately, helping them to not only refer suspicious lesions but also manage other lesions in primary care, thus relieving pressure on busy dermatology departments and saving patients from the anxiety of an unnecessary 2WW referral.

Whilst standards of clinical evidence for AI systems should continue to improve, with more emphasis on prospective clinical trials, it is fair to assume that, much like the existing clinical workforce, no AI system will be 100% sensitive in a real-world deployment. Human expertise can be amplified by AI systems, but human decision-makers need to have the domain knowledge and confidence to disagree with such systems when it is necessary.

This counter-intuitively suggests that AI tools are better suited in the hands of clinicians with certain domain knowledge (senior or specialist clinicians) rather than less expert professionals, and this should perhaps be reflected in early deployments. For the specific case of skin cancer, the results suggested that the more clinicians practised dermatological skills, the more they were able to maximize the benefit of the AI systems. 

How the new relationship between healthcare professionals and AI systems will be regulated in the future requires further exploration [31]. The risk of under-or overestimating the usefulness of AI tools during clinical decision making might lead to severe consequences for patients.

Designing safe, explainable, reliable and trustworthy AI systems based on fair, inclusive and unbiased data is a key element supporting the diffusion of such tools in the medical field. However, medical professionals will need to adapt, learn and put in place behaviour and strategies to accommodate the unavoidable uncertainties around the interaction with intelligent systems. In this sense, the diffusion and adoption of AI in clinical practice will inevitably impact the training and education of clinicians, who should learn how to interact with these systems, establish a practice to minimise and prevent system failure and learn how to operate when the system fails, misbehaves or malfunctions. 

## Figures and Tables

**Table 1:** Statistics for GP performance before and after receiving the fictitious AI assessment.

Decision Making Groups	Before AI			After AI		
	Correct Diagnosis (%)	Correct Management (%)	GP Confidence (%)	Correct Diagnosis (%)	Correct Management (%)	GP Confidence (%)
EC	73.6	82.4	66.8	86.8	90	79.5
Only benign	68	62	63.5	89	84	82.7
Only malignant	77.4	96	69.1	85.4	96	76.5
CU	37.5	44	61.8	54	62.5	63.3
Only benign	9	8	61.7	42	41	62.5
Only malignant	66	80	62.5	66	84	65
DS	46	54	60	22	25	65.7
Only benign	32	32	58.5	10	4	67
Only malignant	60	76	62.5	34	46	64

## Data Availability

The data are not publicly available to protect the privacy and confidentiality of study participant.

## Appendix A

Table A1 shows the ten lesions used in the simulation study and their classification.

**Table A1:** The ten cases used in the simulation study.

Lesions			Classification
	Case 2	Case 3	Correct benign
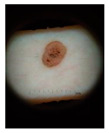
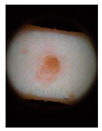
Case 4	Case 5	Case 6	Correct malignant
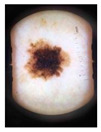
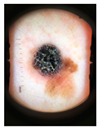
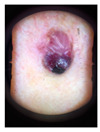
Case 1			Borderline—correct benign
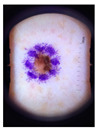
Case 7			Borderline—correct malignant
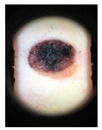
Case 8			Borderline—correct benign
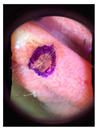
Case 9			Melanoma misclassified as benign
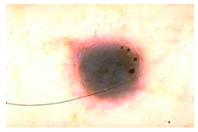
Case 10			Benign misclassified as Melanoma
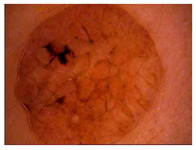

## Appendix B

Computation used to compose the sensitivity indexes:

d’ = z^8^ − z(False)

- Decision wrong before and correct after AI insights = Hit rate
- Decision correct before and wrong after AI insights = False rate
- Decision correct before and correct after AI insights = Correct rejection
- Decision wrong before and wrong after AI insights = Miss
